# Supplementary material for: Plasmids as Key Players in Acinetobacter Adaptation
Source: Int J Mol Sci. 2022 Sep 17;23(18):10893. doi: 10.3390/ijms231810893 (PMC9501444; doi:10.3390/ijms231810893)
Supplement: Supplementary file 1 [file ijms-23-10893-s001.zip › ijms-1920353-supplementary.pdf]

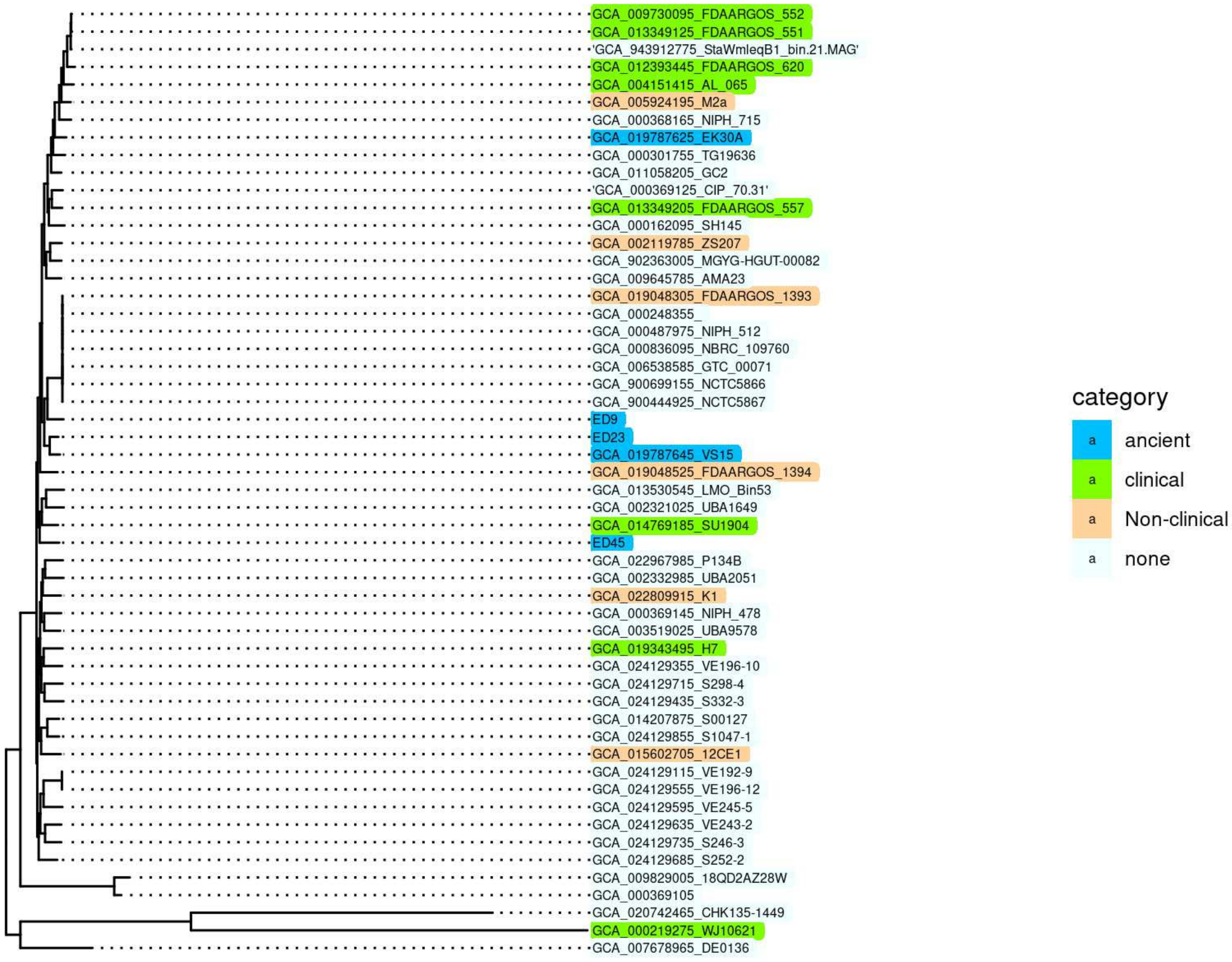

Figure S1. Genome-based phylogeny of *A.lwoffii*. The maximum likelihood tree is based on concatenated nucleotide sequences of single copy genes from all "*Acinetobacter*" genomes. It is clearly seen that the three strains in the lowest part of the tree do not belong to *A.lwoffii*.

Table S1. Clinical strains of *A. lwoffii* used for comparison with environmental strains

Strain	BioProject	Source
SH145	PRJNA38343	Skin
NIPH 715	PRJNA183318	Pus
CIP 70.31	PRJNA183262	Gangrenous lesion
NIPH 478	PRJNA183261	Ear swab
NIPH 512	PRJNA219244	Unknown
TG19636	PRJNA224116	Urine

Table S2. Plasmids from permafrost *A. lwoffii* strains containing genes (operons) of heavy metals and antibiotic resistance

Strain	Plasmid	Size, bp	Heavy metal resistance	Antibiotic resistance genes	Accession number
**ED23-35** permafrost, depth 4,5 m	pALWED1.1	287,631	*mer, chr, czc, nreB*	*tet (H)*	CP082144.1
	pALWED1. 3	16,071	*chrBA*	–	CP082145.1
	**pALWED1.8**	4,135	-	*aadA27*	LN873256.1
**ED43-25** permafrost, depth 2,9 m	pALWED2.1	190,039	*mer, ars, cop*	-	KX426229.1
**ED9-5A** permafrost, depth 6,5 m	pALWED3.6	185,756	*ars, cop, czs*	–	CP032290.2
	pALWED3.1	138,030	*mer, ars, cop, czsx3;czsDx2;nreB*	–	CP083572.1
	pALWED3.5	16,561	*chrAB*	-	CP083573.1
**VS15** permafrost, depth 34,0 m	pALWVS1.1	134,096	*cop, czsA, czcD*x2	–	CP080577.1
	**pALWED1.8**	4,135	-	*aadA27*	LN873256.1
**EK30a** permafrost, depth 47,9 m	pALWEK1.1	209,982	*cop, czcA,czcD* x2	–	CP032102.1
	pALWEK1.4	8,635	–	*cflA*	CP032107.1
	pALWEK1.5	8,227	*chrBA*	–	CP080639.1
	**pALWED1.8**	4,135		*aadA27*	LN873256.1

Table S3. Plasmids from modern *A. lwoffii* strains containing genes (operons) of heavy metals and antibiotic resistance

Strain	Plasmid	Size (bp)	Heavy metal resistance	Antibiotic resistance Genes	Accession number
**12CE1** Australia, digestive tract of Penaeus plebejus	pR4WN_12 CE1	270906	*cop*	*sul1,qacE, aac(6')-1b4*	MT742180.1
**FDAARG0S_1393** Germany, culture_collection	unnamed2	221423	*cop, czc, arsHBC*	-	CP077338.1
	unnamed3	55306	*chrAB,arsCBH, merEDQCPTR*	-	CP077339.1
**FDAARG0S_1394** Germany, culture_collection	unnamed1	121296	*cop, czc*	-	CP077370.1
**ZS207** Poland, microbial mats from Zloty Stok gold mine	pMZS	186588	*arsHBC;* CusA/CzcA	-	CP019144.2
**M2a** Hungary, honey	pAVAci98	>27622	*cop, arsHBC, chr, mer, czc**	-	MK993303
**AL_065-1** Pakistan, bedside rail in hospital intensive care unit	pAL_065-2	284,005	-	APH(3''-V1b GNAT, *sul1, qacE, aac(6')-1b4, arr3, blaNDM-ble, sul2-aph(3')-1bx2; bla NDM, ble, floR*; *aph(6)-1dx2; msr(E)-mph(E); aac(3)-2d*	CP078046.1
	pAL_065-3	158,191	*cop,* CusA/CzcA	-	CP078047.1
	pAL_065-5	13776	*chrBA*	-	CP078049.1
**H7** China, chickens	*pH7-250	250175	-	APH(3')VIb, GNAT, *sul1x2,, qacE, aac(6')-1b4, arr3,, floR, msr(E))-mph(E)*	CP072550.1
	pH7-68	68402	*cop x2,czc, arsHBAC*	-	CP072552.1
	pH7-48	48843		*cmlAfloR*	CP072553.1
**SU1904,** Japan, Homo sapience	pSU1904NDM	43651	-	*bleMBL,blaNDM-1; aphA6*	LC537594.1
**JN49-1,** China, Homo sapience	pNDM-JN01	41084	-	*bleMBL, blaNDM-1; aphA6*	KM210086.1
**WJ10621** China, clinic	pNDM-BJ01	47274	-	*bleMBL, blaNDM-1; aphA6*	JQ001791.1
**FDAARG0S_551** USA, Homo sapience	unnamed1	208308	*cop, czcA*	-	CP054821.1
	unnamed4	7854	*chrBA*	-	CP054825.1
**FDAARG0S_552** USA, Homo sapience	unnamed1	221520	*cop, czcA*	-	CP046295.1
**FDAARG0S_557** USA, Homo sapience	unnamed1	230914	*cop, czsA*	-	CP054804.1
**FDAARG0S_620** USA, Homo sapience	unnamed1	198971	*cop, czsA*	-	JAAXYZ010000001.1
	unnamed2	7739	*chrBA*	-	JAAXYZ010000002.1
